# Study design and rationale of 'Influence of Cilostazol-based triple anti-platelet therapy on ischemic complication after drug-eluting stent implantation (CILON-T)' study: A multicenter randomized trial evaluating the efficacy of Cilostazol on ischemic vascular complications after drug-eluting stent implantation for coronary heart disease

**DOI:** 10.1186/1745-6215-11-87

**Published:** 2010-08-24

**Authors:** Seung-Pyo Lee, Jung-Won Suh, Kyung Woo Park, Hae-Young Lee, Hyun-Jae Kang, Bon-Kwon Koo, In-Ho Chae, Dong-Ju Choi, Seung-Woon Rha, Jang-Whan Bae, Myeong-Chan Cho, Taek-Geun Kwon, Jang-Ho Bae, Hyo-Soo Kim

**Affiliations:** 1 Cardiovascular Center, Seoul National University Hospital, Seoul, Korea; 2 Cardiovascular Center, Seoul National University Bundang Hospital, Seongnam, Gyeonggi-do, Korea; 3 Cardiovascular Center, Korea University Guro Hospital, Seoul, Korea; 4 Chungbuk National University Hospital, Cheongju, Chungcheongbuk-do, Korea; 5 Konyang University Hospital, Daejeon, Korea

## Abstract

**Background:**

Current guidelines recommend dual anti-platelet therapy, aspirin and clopidogrel, for patients treated with drug-eluting stent for coronary heart disease. In a few small trials, addition of cilostazol on dual anti-platelet therapy (triple anti-platelet therapy) showed better late luminal loss. In the real-world unselected patients with coronary heart disease, however, the effect of cilostazol on platelet reactivity and ischemic vascular events after drug-eluting stent implantation has not been tested. It is also controversial whether there is a significant interaction between lipophilic statin and clopidogrel.

**Methods/Design:**

CILON-T trial was a prospective, randomized, open-label, multi-center, near-all-comer trial to demonstrate the superiority of triple anti-platelet therapy to dual anti-platelet therapy in reducing 6 months' major adverse cardiovascular/cerebrovascular events, composite of cardiac death, nonfatal myocardial infarction, target lesion revascularization and ischemic stroke. It also tested whether triple anti-platelet therapy is superior to dual anti-platelet therapy in inhibiting platelet reactivity in patients receiving percutaneous coronary intervention with drug-eluting stent. Total 960 patients were randomized to receive either dual anti-platelet therapy or triple anti-platelet therapy for 6 months and also, randomly stratified to either lipophilic statin (atorvastatin) or non-lipophilic statin (rosuvastatin) indefinitely. Secondary endpoints included all components of major adverse cardiovascular/cerebrovascular events, platelet reactivity as assessed by VerifyNow P2Y12 assay, effect of statin on major adverse cardiovascular/cerebrovascular events, bleeding complications, and albumin-to-creatinine ratio to test the nephroprotective effect of cilostazol. Major adverse cardiovascular/cerebrovascular events will also be checked at 1, 2, and 3 years to test the 'legacy' effect of triple anti-platelet therapy that was prescribed for only 6 months after percutaneous coronary intervention.

**Discussion:**

CILON-T trial will give powerful insight into whether triple anti-platelet therapy is superior to dual anti-platelet therapy in reducing ischemic events and platelet reactivity in the real-world unselected patients treated with drug-eluting stent for coronary heart disease. Also, it will verify the laboratory and clinical significance of drug interaction between lipophilic statin and clopidogrel.

**Trial Registration:**

National Institutes of Health Clinical Trials Registry (ClinicalTrials.gov identifier# NCT00776828).

## Background

Current guidelines recommend at least 6 months of dual anti-platelet agents, consisting of aspirin and a thienopyridine, after percutaneous coronary intervention (PCI) and ideally, 1 year of both drugs [1]. However, some patients experience acute thrombotic events, the incidence of which has been diverse from less than 1% to 3% [2,3] even with good adherence to this dual anti-platelet therapy (DAT), and also, late restenotic complication after PCI. In addition, as interventional cardiologists face more and more complex lesions, the risk of ischemic events, including cardiac events, may tend to rise. These problems raise the issue to whether there are effective additional methods or drugs to prevent ischemic complications.

Cilostazol is a selective phosphodiesterase-3 (PDE-3) inhibitor, which has various effects including vasodilatory, anti-platelet and partially, anti-inflammatory effect. Several investigators have focused on cilostazol as one of the candidate drugs that can reduce ischemic events after PCI. Inspite of the fact that cilostazol is metabolized by CYP 3A [4] and to a lesser degree 2C19 [5], the same pathway as that of clopidogrel, cilostazol was suggested to be effective in reducing platelet reactivity in vitro [6] and also, efficacious and safe in reducing thrombotic complications in the real world [7]. Also, several studies have suggested that cilostazol successfully and safely reduces restenosis rate after PCI [8-11]. In a meta-analysis, cilostazol has shown to be consistently helpful in reducing binary angiographic restenosis and repeat revascularization. But these data were derived mainly from the BMS era [12]. Data from a few randomized trials have suggested that cilostazol reduces late luminal loss also in drug-eluting stents (DES) [10,13]. But, these trials were underpowered to see whether cilostazol reduces the ultimate core endpoint of clinical trials, major adverse cardiac events (MACE) or major adverse cardiovascular/cerebrovascular events (MACCE). And the restricted inclusion criteria of the previous randomized trial cannot answer to the question whether cilostazol would be effective also in a routine clinical practice. These previous reports call for a large, randomized, controlled study to investigate whether cilostazol can really reduce MACE/MACCE after PCI in a real-world practice and also, for an extensive follow-up of these patients to see whether the effects persists in the long-term, as in a recent pooled analysis [14].

Additionally, there have been numerous questions as to whether there are significant interaction between clopidogrel and lipophilic statin and whether such interaction can be overcome by the addition of cilostazol. Although data on this matter is controversial but somewhat been in favor of being no significant in vivo interaction between clopidogrel and lipophilic statin [15], the interpretation and application of these data have been restricted by the unrandomized/unstratified manner of the studies [16-18] and non-prospective collection of the data [19]. Also, although the pleiotrophic effect of cilostazol in renal function has been demonstrated in some animal experiments [20,21], whether this holds true for humans have never been investigated.

In order to provide a definite answer to the above questions, we planned the 'Influence of **CIL**ostazol-based triple anti-platelet therapy **ON **Ischemic Complication after drug-eluting sten**T **implantation (**CILON-T**)' study. In this study, we compared the efficacy and safety of triple anti-platelet therapy (TAT), composed of aspirin, clopidogrel and cilostazol, with the conventional DAT by evaluating MACCE and platelet reactivity in an unselected population of patients receiving PCI with DES for coronary heart disease (CHD) in real-world practice. In addition, we would like to get answers for the issues whether lipophilic statin-clopidogrel interaction would influence the patient outcome and whether cilostazol can overcome such interaction, using atorvastatin (Lipitor^®^, Pfizer) dependent on cytochrome P450-3A4 (CYP3A4) for metabolism versus rosuvastatin (Crestor^®^, AstraZeneca) independent from CYP3A4.

## Methods/Design

### Study objectives, hypothesis and design

**Figure 1:**
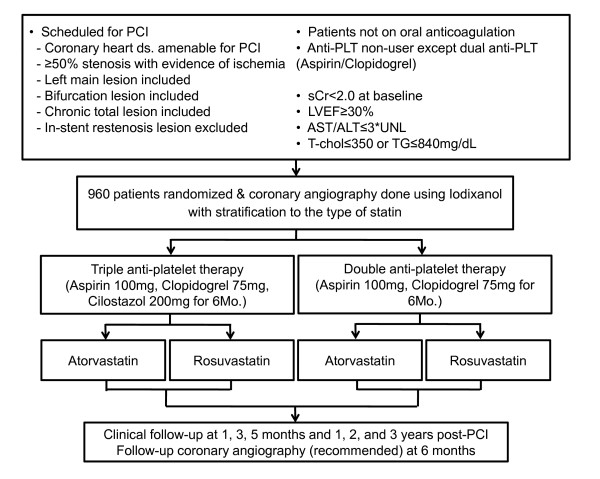
Flowchart of the enrolled patients

The primary objective of this study was to compare the efficacy of TAT with DAT in reducing 6 months' MACCE after PCI in those with documented CHD. The working hypothesis of this trial was 'TAT is superior to DAT in reducing 6 months' MACCE, the composite of cardiac death, nonfatal myocardial infarction (MI), ischemic stroke and target lesion revascularization (TLR) in CHD patients receiving PCI with DES'. The trial also addressed whether TAT is superior to DAT in reducing platelet reactivity and also, whether there is any clinical effect of statin-clopidogrel interaction on MACCE according to the type of statin. The protocol of the trial has been registered at http://www.clinicaltrials.gov (NCT00776828) and a brief flowchart of the whole study is summarized in Figure 1. The endpoints are summarized in Appendix 1.

### Patient population

Patients at least 18 years of age who had typical ischemic symptoms or positive stress test and a native coronary lesion (≥50% diameter stenosis by visual estimation on coronary angiogram and reference diameter≥2.5 mm)) were included in this study. Five high-volume centers in Korea participated and enrolled the patients in this multicenter, open-label, prospective, randomized trial. There were nearly no angiographic limitations about the number of lesions, vessels, location of lesions or their length, to investigate the effect of cilostazol in the real-world setting. The following patients were excluded from the study; cardiogenic shock; explicit side effect or contraindications to anti-platelet agents including cilostazol; concomitant treatment with any other anti-platelet agent or anticoagulant other than the study drugs; current user of cilostazol before the entry of study. The detailed inclusion and exclusion criteria are summarized in Appendix 2.

### Randomization and interventions

All patients received 300 mg aspirin and 300~600 mg clopidogrel loading before the procedure unless the patient had been taking these medications for at least 1 week before the procedure. Patients were randomized to receive either conventional DAT or TAT and also, randomly stratified to take equipotent doses of either atorvastatin 20 mg or rosuvastatin 10 mg per day before the interventional procedure. The patients were also stratified according to the enrolling medical centers/hospitals. A loading dose of cilostazol (Otsuka Korea) 200 mg was given immediately before the procedure. Random allocation of the patients was done via a Web-based computerized program separately managed at medical research collaboration center, Seoul National University Hospital. PCI was done according to the standard technique and the decision of the pre-dilatation and use of glycoprotein IIb/IIIa inhibitors were up to the operator's discretion. The target lesion/vessel was designated as the first lesion/vessel that was intervened. Platelet reactivity was measured by using the Ultegra Rapid Platelet Function Assay (VerifyNow^® ^Aspirin and VerifyNow^® ^P2Y12 assay, Accumetrics Inc., San Diego, California, USA) immediately before discharge.

After enrollment and index PCI procedure, clinical follow-up was planned at 1, 3, 6 months and all patients were recommended follow-up coronary angiography at 6 months according to local clinical practice. Platelet reactivity was also measured at 6 months after index procedure. The investigators were urged to follow the patients, either by office visits or by telephone contacts as necessary. Patient adherence to the study drug was checked at every outpatient visit and the decision of the drug discontinuation was always discussed and checked under the physician's recommendation.

### Outcome measures

The primary endpoint of this study was the rate of MACCE, a composite ischemic vascular event following PCI, including cardiac death, nonfatal MI, TLR and ischemic stroke at 6 months in the DAT and TAT group. The key secondary endpoints defined a priori are

- P2Y12 reaction unit (PRU) as assessed by VerifyNow^® ^P2Y12 assay

- Cardiac death, nonfatal MI, TLR, target vessel revascularization (TVR), stent thrombosis and ischemic stroke

- MACCE according to the type of statin and its interaction with clopidogrel

- Bleeding, as defined according to the TIMI bleeding classifications [22], major bleeding defined by the presence of at least 1 of the following: intracranial bleeding (documented with magnetic resonance imaging, computed tomography, any other examination or autopsy), or clinically overt bleeding resulting in a 5 g/dL decrease in hemoglobin value (or, when hemoglobin values were not available, a 15% decrease in hematocrit).

- Angiographic outcome, such as late luminal loss, binary restenosis at 6 months' follow-up angiography

- Albumin-to-creatinine ratio

### Statistical Analysis

#### Sample size calculation

To test the hypothesis that TAT is superior to DAT in reducing MACCE at 6 months after PCI, based on the previous outcome data [10,13,23], we assumed that MACCE rate in DAT and TAT group would be 10% and 5% respectively. Using a superiority design, an estimated total of 960 patients was needed to ensure a power of at least 80% to detect a 5% difference in the composite of ischemic vascular events between the two anti-platelet therapy groups using a two-sided t-test, with a sampling ratio of DAT:TAT at 1:1, bilateral risk set at 5% and drop-out rate estimated to be 10%.

#### Statistical Analyses

All analysis will be done by intention-to-treat method (all patients analyzed as part of their assigned treatment group). However, per protocol (patients analyzed as part of their assigned group only if they actually received their assigned treatment) and as-treated analysis will also be performed solely for descriptive comparison.

The primary endpoint of 6 months' MACCE will be analyzed using χ^2^-test on an intention-to-treat analysis. The hypothesis will be evaluated based on a superiority testing. The clinical secondary endpoints will also be tested based on χ^2^-test, similarly as primary endpoint testing. The endpoints will be analyzed in pre-specified subgroups, which includes the presence of diabetes mellitus, long lesions (lesion length≥28 mm), small vessel lesions (lesion diameter < 2.75 mm), age and sex. Also, most importantly, post-hoc analysis of MACCE according to the type of statin given (atorvastatin vs. rosuvastatin) will be done. Analysis of platelet function will be tested using repeated ANOVA method with the Bonferroni's correction.

Continuous variables will be presented as mean ± SD and compared with the use of unpaired Student's t-test or in the case of non-normal distribution, Mann-Whitney U test. Categorical variables will be summarized as number or percentages and compared with χ^2 ^or Fisher's exact test, as appropriate. Survival curves using all available follow-up data will also be constructed for time-to-event variables using Kaplan-Meier estimates and compared by log-rank test. A p-value < 0.05 will be considered statistically significant.

### Trial Organization

#### Executive Committee

The Executive Committee, constituted of the study chairperson and the principal investigators of the investigating centers, approved the final trial design and protocol issued to the Data and Safety Monitoring Board (DSMB) and the clinical sites. In addition, it was also responsible for reviewing the results, determining the adjudication of the publication, and selection of secondary projects by members of the Steering Committee.

#### Data Safety Monitoring Board (DSMB)

An independent DSMB, composed of general and interventional cardiologists, and a biostatistician abided by the applicable regulatory guidelines and did not participate in the trial. The DSMB committee reviewed the safety data from this study and made recommendations, based on safety analyses of unanticipated device effects (UADEs), serious adverse events (SAEs), protocol deviation, device failures, and follow-up reports. In addition to the scheduled DSMB meetings, that were determined prior to the initiation of the study, the board convened a meeting at any time if safety problems became an issue. The DSMB were responsible for recommending the Executive Committee to modify or stop the study if there were any safety or compliance issue. However, the final decisions regarding study modifications rested with the Executive Committee. Cumulative safety data were reported to the DSMB and reviewed on an ongoing basis throughout enrollment and follow-up to ensure safety of the patients. Every effort was made to allow the DSMB to conduct an unbiased review of the patients' safety information. All DSMB reports were made available to the appropriate agencies upon request but were otherwise remained strictly confidential. Prior to the DSMB's first review of the data, the DSMB charter was drafted. The plan defined the stopping rules for stopping the trial for safety. The first meeting of the DSMB was requested for discussion of the protocol and an understanding of all the protocol elements. The DSMB developed a consensus understanding of all trial endpoints and definitions used in the event adjudication process.

#### Clinical Event Adjudication Committee (CEAC)

The Clinical Events Adjudication Committee (CEAC) was comprised of interventional and non-interventional cardiologists who were not participants in the study. The CEAC was responsible for the development of specific criteria used for categorization of clinical events and clinical endpoints in the study, which were based on protocol. At the onset of the trial, the CEAC established clear rules stating the minimum amount of data required, and the algorithm followed in order to classify a clinical event. All members of the CEAC were blinded to the primary results of the trial and met regularly to review and adjudicate all clinical events in which the required minimum data was available. The Committee also reviewed and ruled on all deaths that occurred throughout the trial.

#### Data Coordination and Site Management

Data coordination and site management services were performed by the Clinical Trials Center at Seoul National University Hospital.

#### Ethical approval

This study was approved by institutional review board of Seoul National University Hospital.

## Discussion

With the advent of various coronary balloons, stents and wires, PCI is widely used and accepted not only to improve angina symptoms but also to reduce cardiovascular mortality, albeit in the setting of acute coronary syndrome. Although the development of DES has been a major leap in the field of interventional cardiology in reducing recurrent ischemic events, it has been mandatory to use DAT for at least 6 months and preferably, 1 year to prevent stent thrombosis (ST). Even under the protection of DAT, some subsets of patients suffer from cardiovascular ischemic events including ST, necessitating search for new methods to prevent future ischemic cardiovascular events.

### Rationale of cilostazol use to prevent ischemic cardiovascular events

Cilostazol is a selective PDE3 inhibitor that prevents degradation of intracellular cAMP into 5'-AMP. It has various action mechanisms on various cells. For example, cilostazol has been shown to inhibit the expression of platelet activation markers, such as p-selectin [24] and glycoprotein IIb/IIIa receptors, thus inhibiting platelet aggregation and adhesion [25]. It has also been proved that cilostazol enhances endothelial function by nitric oxide production [26] and reduces various inflammatory responses in endothelial cells [27]. Furthermore, cilostazol is pro-apoptotic to vascular smooth muscle cells and ultimately reduces neointimal formation [28]. Taken together, cilostazol is a drug that may prevent ST, restenosis and improve endothelial function theoretically.

### Previous reports of cilostazol use in PCI and the rationale of the CILON-T trial

With the potential benefit of cilostazol on vascular function in vitro, there have been several previous efforts to prove the efficacy of cilostazol in patients undergoing PCI. For example, the efficacy of cilostazol has been tested in patients who received percutaneous transluminal coronary angioplasty (PTCA) [29], bare-metal stenting [7,9,30] and also in those at high risk of events after DES implantation [10,13]. A recent systematic review of the above mentioned trials utilizing adjunctive cilostazol has reported an appraisal of cilostazol for inhibiting neointimal hyperplasia [12]. However, very few trials have effectively nor properly addressed the issue of whether cilostazol can reduce MAC(C)E, the ultimate concern of all trials. Especially, this issue has never been addressed in the DES era nor in the situation of real-world practice, urging a need for a randomized clinical trial to answer this question.

### Issues sought in the CILON-T trial

In addition to the primary endpoint of whether TAT would be superior to DAT in reducing the ischemic vascular events after PCI, CILON-T trial is planned to assess several issues that remains to be answered. The issue of whether laboratory benefits are directly connected with clinical outcomes will be sought. Specifically, there have been papers demonstrating that PRU levels predicted adverse cardiovascular events in high risk patients [31-33] and that cilostazol reduced the average level of platelet activation in patients with coronary heart disease [6] and also, PRU level [34]. However, racial difference is one factor that can influence the platelet reactivity [35] and there remains a paradox between the higher level of PRU and lower rate of thrombotic events after DES implantation in Asians. Simply compared, the rate of stent thrombosis involving more than 3,000 patients around 1.5 years after DES implantation is 1.9% in Europeans [36] while 0.77% in Japanese [2]. In this CILON-T trial, we can get valuable information whether cilostazol-induced reduction of PRU levels would lead to beneficial clinical outcomes.

It is also expected to shed light on whether non-CYP3A4-metabolized statin is superior to CYP3A4-metabolized statin in terms of MAC(C)E and if so, whether cilostazol would be effective in reducing this difference. In vitro studies have persistently suggested that the effect of clopidogrel would be reduced by concomitant use of lipophilic statin [37,38] but large in vivo studies do not show trend toward detrimental effect of these types of statin [16-18,39]. However, these in vivo results come from observational registry data or post-hoc analysis of randomized clinical trials. Therefore, our data would add additional information to clopidogrel-statin interaction issue and also, if any, a new finding on the effect of cilostazol in this drug interaction.

In conclusion, we still do not know whether TAT is superior to DAT in reducing MACCE after DES implantation for coronary heart disease in the unselected real-world patients. Furthermore, whether cilostazol-induced PRU level reduction will turn out to improve clinical outcomes has never been elucidated. We hope to address these issues in the CILON-T trial where we enrolled a large unselected population of patients treated with DES for significant coronary heart disease.

## Competing interests

The authors declare that they have no competing interests.

## Authors' contributions

SPL contributed to the design, acquisition of the data, critical establishment/management of the whole database and also, wrote the manuscript. JWS contributed to the design, acquisition and interpretation of the data and critical establishment/management of the whole database.

KWP involved in drafting of the manuscript and critical revision of the study design. HYL contributed to the critical acquisition of the data and initial conception of the study design.

HJK contributed to the critical acquisition of the data and initial conception of the study design. BKK contributed to the critical acquisition of the data and initial conception of the study design. IHC contributed to the critical acquisition of the data and initial conception of the study design. DJC contributed to the critical acquisition of the data and critical reading/revision of the study manuscript. SWR contributed to the critical acquisition of the data and initial conception of the study design. JWB contributed to the critical acquisition of the data. MCC contributed to the critical acquisition of the data and initial conception of the study design. TGK contributed to the critical acquisition of the data. JHB contributed to the initial conception and design of the whole study, critical acquisition of the data and also, final approval of the version of the manuscript. HSK contributed to the initial conception and design of the whole study, critical acquisition of the data and also, final approval of the version of the manuscript.

## Appendix

### Appendix 1. Endpoints of CILON-T trial

#### Primary endpoints

MACCE (composite of cardiac death, nonfatal MI, TLR and ischemic stroke)

#### Secondary endpoints

Individual composites of MACCE

Platelet reactivity unit as assessed by Ultegra Rapid Platelet Function Assay (VerifyNow^®^)

MACCE according to the type of statin and its interaction with clopidogrel

Bleeding complications, as defined by TIMI classification

Angiographical outcome at 6 months' angiographical follow-up (late luminal loss, binary restenosis)

Albumin-to-creatinine ratio

### Appendix 2. Enrollment criteria for CILON-T trial

#### General inclusion criteria

At least 18 years of age

Able to confirm and understand the risk, benefits and treatment alternatives of receiving cilostazol and

also to provide written informed consent

Significant coronary artery stenosis (≥50% by visual estimate)

Evidence of myocardial ischemia (stable angina, acute coronary syndrome with evidence of myocardial

ischemia by symptomatic assessment or by stress test)

Adequate candidate for percutaneous coronary intervention (PCI)

#### General exclusion criteria

Known hypersensitivity or contraindications to heparin, aspirin, clopidogrel, contrast media, cilostazol or statin

Previous anti-platelet user (excluding aspirin or clopidogrel) or warfarin user

Female of childbearing potential, unless a recent pregnancy test is negative, who possibly plans to become pregnant in the near future

History of bleeding diasthesis or coagulopathy or baseline thrombocytopenia (PLT count < 120,000/μL)

Chronic kidney disease (defined as Cr≥2.0 mg/dL or on dialysis)

Poor left ventricular systolic function (LVEF < 30%)

Significant liver disease (defined as AST/ALT > 3×UNL or liver cirrhosis Child B/C)

Significant dyslipidemia (total cholesterol≥350 mg/dL or triglyceride≥840 mg/dL)

Age > 80

Chronic alcohol use or drug abuser

#### Angiographic inclusion criteria

Target lesion(s) located in a native coronary artery with visually estimated diameter≥2.5 mm

Target lesion(s) amenable to PCI (left main, bifurcation and chronic total occlusion lesion included)

#### Angiographic exclusion criteria

Target lesion(s) with ISR
